# Derivation and validation of murine histologic alterations resembling asthma, with two proposed histologic grade parameters

**DOI:** 10.1186/1471-2172-10-58

**Published:** 2009-10-30

**Authors:** Mitchell S Wachtel, Goutam Shome, Mhairi Sutherland, John J McGlone

**Affiliations:** 1 Department of Pathology, Texas Tech University Health Sciences Center, Lubbock, Texas, USA; 2 Department of Internal Medicine, Texas Tech University Health Sciences Center, Lubbock, Texas, USA; 3 Department of Animal and Food Sciences, Texas Tech University, Lubbock, Texas, USA; 4 Animal Care Services, Texas Tech University, Lubbock, Texas, USA

## Abstract

**Background:**

The objective was to define murine histologic alterations resembling asthma in a BALB/c OVA model and to suggest grading criteria. Identified were six salient histologic findings in lungs with putative allergic inflammation: 1) bronchoarterial space inflammation; 2) peri-venular inflammation; 3) inflammation about amuscular blood vessels; 4) inter-alveolar space inflammation, not about capillaries; 5) pleural inflammation; and 6) eosinophils within the inflammatory aggregates. An initial study comprised six groups of twelve mice each: 1) stressed, control; 2) stressed, sensitized; 3) stressed, challenged; 4) not physically stressed, control; 5) not physically stressed, sensitized; 6) not physically stressed, challenged. A second study comprised four experimental groups of twenty mice each: 1) stressed, control; 2) stressed, challenged; 3) not physically stressed, control; 4) not physically stressed, challenged. A third study evaluated two grading criteria, 1) the proportion of non-tracheal respiratory passages with inflammatory aggregates and 2) mitoses in the largest two non-tracheal respiratory passages, in five groups of five mice each, evaluated at different times after the last exposure.

**Results:**

The first study suggested the six histological findings might reliably indicate the presence of alterations resembling asthma: whereas 82.4% of mice with a complete response had detectable interleukin (IL)-5, only 3.8% of mice without one did; whereas 77.8% of mice with a complete response were challenged mice, only 6.7% of mice without complete responses were. The second study revealed that the six histological findings provided a definition that was 97.4% sensitive and 100% specific. The third study found that the odds of a bronchial passage's having inflammation declined 1) when mitoses were present (OR = 0.73, 0.60 - 0.90), and 2) with one day increased time (OR = 0.75, 0.65 - 0.86).

**Conclusion:**

A definition of murine histologic alterations resembling asthma in the BALB/c OVA mouse was developed and validated. The definition will be of use in experiments involving this model to ensure that all mice said to have undergone an asthmatic attack did indeed reveal allergic pulmonary inflammation. Proposed grading criteria should be further evaluated with additional studies using physiologic measures of attack severity and increased airway resistance.

## Background

Asthma currently afflicts about 300,000,000 persons and was estimated in 2005 to have killed 255,000 [1]. Although some have considered murine models to be inappropriate as means of understanding human asthma [2], most feel the mouse model is vital, especially with respect to the understanding of the early response to antigens and the mediators of allergic asthma [3,4]. The BALB/c OVA mouse model is one of the most commonly used [5]. Although a general understanding of the histological findings of the BALB/c OVA mouse has been generated by the publication of different studies [3], a systematic approach to the most important aberrations to garner a readily identifiable histological definition has not been performed. Such a definition would be of utility as a safeguard when performing studies using the BALB/c OVA mouse because it would ensure that all individual animals submitted for study experienced histologic changes resembling asthma. Moreover, such a definition would be of use because therapeutic manipulations that abolish the findings would then be designated as successfully obliterating the attack. Posited was a specific pattern of histological findings, derivable from generally accepted observations of BALB/c OVA mice. Six salient findings were identified in lung sections with putative allergic inflammation: 1) bronchoarterial space inflammation; 2) peri-venular inflammation; 3) inflammation about amuscular blood vessels; 4) inter-alveolar space inflammation, not about capillaries; 5) pleural inflammation; and 6) eosinophils within the inflammatory aggregates. An initial study comprised of six experimental groups of twelve mice each, randomly assigned: 1) stressed, control; 2) stressed, sensitized; 3) stressed, challenged; 4) not physically stressed, control; 5) not physically stressed, sensitized; 6) not physically stressed, challenged. A second study comprised of four experimental groups of twenty mice each, randomly assigned: 1) stressed, control; 2) stressed, challenged; 3) not physically stressed, control; 4) not physically stressed, challenged. A third study evaluated two grading criteria, 1) the proportion of non-tracheal respiratory passages with inflammatory aggregates and 2) the presence or absence of mitoses in the largest two non-tracheal respiratory passages, in five experimental groups of five mice each, assigned to different times after the last exposure.

## Results

### Definition of murine histologic alterations resembling asthma

**Figure 1:**
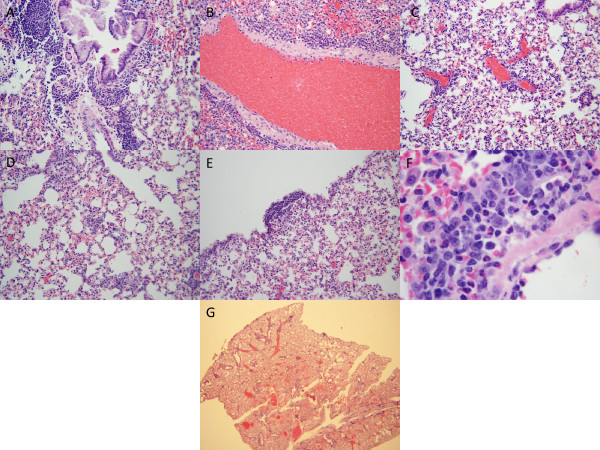
**Photomicrographs of mouse lungs with a complete response**. a. Inflammation in bronchoarterial space (hematoxylin and eosin, original maginification 200 ×). b. Inflamation about pulmonary vein (hematoxylin and eosin, original maginification 200 ×); c. inflammation about amuscular blood vessels (hematoxylin and eosin, original maginification 200 ×); d. Inflammation in inter-alveolar spaces, not surrounding blood vessels (hematoxylin and eosin, original maginification 200 ×); e. Pleural inflammation (hematoxylin and eosin, original maginification 200 ×); f. Eosinophils within inflammatory aggregate (hematoxylin and eosin, original maginification 1000 ×); g. Low power appearance (hematoxylin and eosin, original magnification 40 ×).

**Figure 2:**
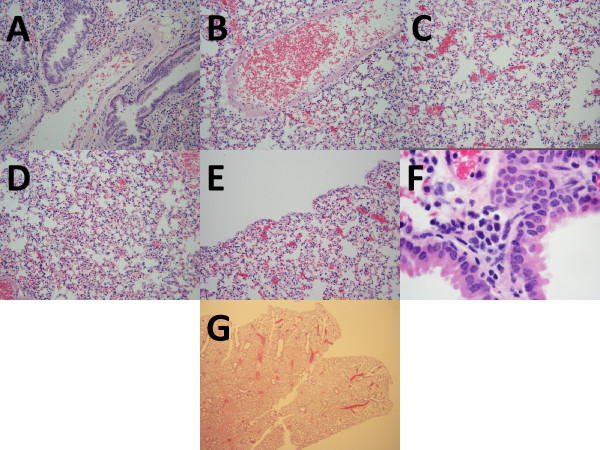
**Photomicrographs of control mouse lungs**. a. Bronchoarterial space without inflammation (hematoxylin and eosin, original maginification 200 ×). b. Pulmonary vein without inflammation (hematoxylin and eosin, original maginification 200 ×); c. Amuscular blood vessels without inflammation (hematoxylin and eosin, original maginification 200 ×); d. Inter-alveolar spaces without inflammation (hematoxylin and eosin, original maginification 200 ×); e. Pleura lacking inflammation. (hematoxylin and eosin, original maginification 200 ×); f. Small peri-bronchial inflammatory aggregate without eosinophils (hematoxylin and eosin, original maginification 1000 ×); g. Low power appearance (hematoxylin and eosin, original magnification 40 ×).

Based on evaluation of prior literature [6-26] and detailed examination of associated photomicrographs, six histological features were deemed most important and most reliably assessed: 1) inflammation of bronchoarterial spaces; 2) perivenular inflammation; 3) inflammation about amuscular blood vessels; 4) inflammation in inter-alveolar spaces, not surrounding capillaries; 5) pleural inflammation; and 6) eosinophils within the inflammatory aggregates. Each of the features is shown in Figure 1; corresponding photographs of normal mouse lungs are shown in Figure 2. For the first study set, mice lungs were assessed for each characteristic at 200×, before the mice were divided into complete response (all six changes), incomplete response (1 to 6 changes), and no response (0 changes). The presence of all six findings indicated that inflammatory infiltrates were distributed throughout the lung; further subclassification of location was impossible for routine assessments because, for any particular section, the size of the respiratory passages and pulmonary veins and their location with respect to the trachea and pleura depend upon the precise location of the section within the lung and the orientation of the lung at the time histological sections are created, matters that cannot be controlled without great difficulty. There were 27 mice with a complete response, 29 mice with an incomplete response, and 16 mice with no response. Attempts to use a continuous variable for the histologic changes (e.g., the number of characteristics) yielded models that fit poorly; moreover, as shown below there was excellent correlation between allergic challenge and complete response and good correlation between incomplete response and sensitization and between no response and control. With a modest degree of experience, complete responses were distinguished in seconds at low power; photomicrographs of a complete response at low power (fig. six-g) and a normal lung at low power (fig. seven - g) are provided. Evaluations for the second and third study sets began with evaluation at 20×, always confirmed at high power for the presence of eosinophils in the infiltrates.

### Studies to establish and validate the definition of murine histologic alterations resembling asthma

**Figure 3:**
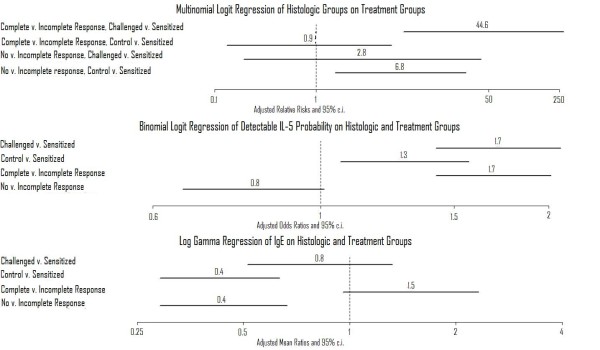
**Initial study**. Results of regression analyses performed to evaluate the relationship of the differing immunologic treatments to the differing histologic categories (top) and the relationship of the differing histologic findings and immunologic treatments to IL-5 detectability (bottom).

Of the 72 mice in the first study, BAL fluid from 47 were evaluated for detectable interleukin (IL)-4 and 43 were assayed for detectable IL-5, and serum from 61 mice were examined for the IgE concentration. A table [see Additional file 1- Table S1] shows that differences in proportions with respect to histological groups and 1) immunologic groups, 2) detectable IL-5, and 3) Immunoglobulin E (IgE), and with respect to immunologic groups and 1) detectable IL-5 and 2) IgE could not be explained by chance (*P *< 0.05 for each examination). Differences in proportions with respect to IL-4 and 1) histological groups and 2) treatment could have been explained by chance (*P *> 0.05 for each examination). Differences in proportions with respect to Stress and 1) histological groups, 2) immunologic groups, 3) detectable IL-4, 4) detectable IL-5, and 6) IgE could have been explained by chance (*P *> 0.05 for each examination). Multinomial logit regression of histological groups performed with forward step regression with BIC identified only immunologic groups as being important; the top half of figure 3 displays the results. The ratio of mice with a complete response to mice to mice with an incomplete response was about 45 times greater for challenged than for immunized mice (*P *< 0.05); differences between control and sensitized mice might have been due to chance (*P *> 0.05). The ratio of mice with no histological response to mice to mice with an incomplete histological response was about 7 times greater for control than for sensitized mice (*P *< 0.05); differences between challenged and sensitized mice might have been due to chance (*P *> 0.05). Binomial logit regression of detectable IL-5 performed with forward step regression with BIC identified immunologic groups and histological groups as being important; the bottom half of figure 3 displays the results. Adjusted for histological group, the odds of a challenged mice having detectable IL-5 are about 70% greater than those of a sensitized mouse (*P *< 0.05). Adjusted for histological group, the odds of a control mouse's having detectable IL-5 are about 30% greater than those of a sensitized mouse (*P *< 0.05); both control mice who had detectable IL-5 had complete responses. Adjusted for immunologic treatment group, the odds of a mouse with a complete response's having detectable IL-5 are about 70% greater than those of a mouse with an incomplete response (*P *< 0.05); differences between mice with no response and mice with incomplete responses might have been due to chance (*P *> 0.05). Log gamma regression of IgE concentration performed with forward step regression with BIC identified immunologic groups and histological groups as being important; the bottom half of figure 3 displays the results. Adjusted for histological group, the odds of a challenged mouse's having detectable IL-5 are about 70% greater than those of a sensitized mouse (*P *< 0.05). Adjusted for histological group, control mice IgE levels are about 60% less than are those of sensitized mice (*P *< 0.05); differences between challenged mice and sensitized mice might have been due to chance (*P *< 0.05). Adjusted for immunologic treatment group, mice with no response had about 60% less IgE than mice with an incomplete response (*P *< 0.05); differences between mice with incomplete responses and mice with complete responses might have been due to chance (*P *> 0.05). The results indicate that sensitization correlated with an elevation in IgE that was not changed by allergic challenge more than would be expected by chance, validating its utility as a control. Taken together, the results suggest a complete response might be a good definition of the histologic alterations resembling asthma: whereas 82.4% of mice with a complete response had detectable IL-5, only 3.8% of mice without one did (OR 93.4, 95% c.i. 9.5 - 4904.8, *P *< 0.05); whereas 77.8% of mice with a complete response were challenged mice, only 6.7% of mice without complete responses were (OR 44.6, 9.6 - 304.7, *P *< 0.05).

Of the 80 mice from the second study, two mice, both challenged and stressed, expired before the end of the swim stress; the pair was excluded from further assessment. A second table [see Additional file 2- Table S2] shows that the different experimental protocols differentiated mice with respect to the presence or absence of a complete response protocols (*P *< 0.05) and that they failed to do so with respect to hemorrhage (*P *> 0.05) and alveolar dilatation (*P *> 0.05). Stress did not affect the likelihood of a complete response; complete responses were seen in 18 of 38 (47.4%) stressed mice and 19 of 40 (47.5%) non-stressed mice (*P *> 0.05). By contrast, complete responses were seen in 37 of 38 (97.4%) challenged mice and 0 of 40 (0%) control mice (*P *< 0.05), yielding a sensitivity of 97.4% and a specificity of 100%.

### Studies performed to propose potential grading parameters

**Figure 4:**
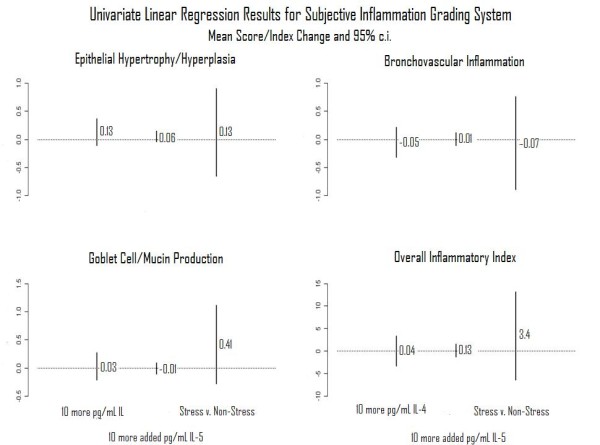
**Semi-quantitative inflammatory grading system**. Results of univariate regression analyses performed to evaluate relationships between each of three predictor variables, IL-4, IL-5, and stress, and each of four outcome variables, epithelial hypertrophy/hyperplasia, bronchovascular inflammation, goblet cell/mucin production, and the overall inflammatory index.

For the evaluation of the semi-quantitative inflammation grading system, all 27 complete response mice from the first study set were used. Eighteen had evaluable IL-4 measurements; 17 had evaluable IL-5 measurements. Figure 4 displays results of univariate regressions on the modified subjective inflammatory grading system. All evaluated relationships could have been explained by chance (*P *> 0.05 for each analysis). Goblet cell/mucin production had the largest effect size with respect to stress, but in the opposite direction that had been indicated by Pastva [17] and then in a fashion explainable by chance (*P *> 0.05). Proliferation and inflammation had opposed effects explainable by chance with respect to stress and detectable IL-4.

A third table [see Additional file 3- Table S3] displays results of the study of the quantitative proliferation/hypertrophy markers that evaluated 64 mice from the second study set. Relationships of cells per 0.1 mm and mitoses with dilatation, hemorrhage, stress and one another were explainable by chance (*P *> 0.05 for each evaluation). The median number of cells per 0.1 mm of basement membrane was four fewer for allergic mice than control mice (*P *< 0.05); the difference, too small to use for evaluation of subgroups of challenged animals, was consistent with the presence of larger cells, perhaps via induction of goblet cell metaplasia. Mitoses were seen in 65.6% of allergic mice and 3.1% of controls (OR = 54.9, 7.3 - 2484.5, *P *< 0.05).

**Figure 5:**
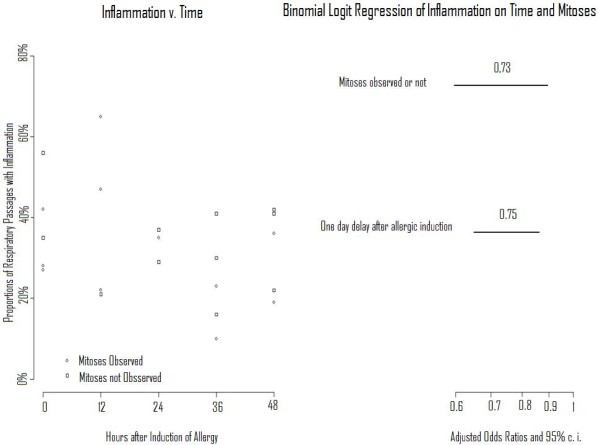
**Study of mice euthanized at different times after challenge**. Scatterplot of proportions of respiratory passages with inflammation and times after treatment (left). Results of regression analysis performed to evaluate the relationship of mitotic activity and time after treatment to the proportion of respiratory passages with inflammation (right).

Of 25 mice in the third study set, two lacked a complete histological response, one at 12 and one at 24 hours. Figure 5 presents the results of evaluations of the remaining 23 mice. On the left is a scatter plot of proportions of respiratory passages with chronic inflammatory infiltrates and times after exposure, with presence or absence of mitoses differentiated by symbols. On the right are results of step forward binomial logit regression; the odds of a bronchial passage's having inflammation declined 1) when mitoses were present (OR = 0.73, 0.60 - 0.90, *P *< 0.05), adjusted for time, and 2) with increased time (OR with one day of increased time = 0.75, 0.65 - 0.86, *P *< 0.05), adjusted for mitoses.

## Discussion

Histologic changes deemed important in the OVA BALB/c mouse model in previous studies were found in the current effort, but this study identified a set of six histological findings that the second study set showed was 97.4% sensitive and 100% specific for mice underwent an allergic challenge. Acute stress was evaluated by multiple comparisons and never found to be of importance in this model. Alveolar dilatation and hemorrhage, not being shown to be associated with allergic challenge, stress, detectable IL-4, detectable IL-5, epithelial cell concentration, or mitotic activity, likely resulted from bronchoalveolar lavage (BAL). Although the semi-quantitative grading system did not prove successful in the first study group, quantitative measures proved more promising: in the second study group, challenged mice, when compared with control mice, had a small but significant decrease in bronchial epithelial cells per 0.1 mm basement membrane and markedly increased odds of having mitoses in the largest non-tracheal respiratory passage. In the third study, time since exposure was associated with a decline in the proportion of inflamed non-tracheal respiratory passages, a decline that was increased by the presence of mitoses in the two largest non-tracheal respiratory passages. The latter finding allows one to posit as grading parameters the proportion of inflamed respiratory passages and mitotic activity.

Prior studies of the BALB/c OVA mouse asthma model that evaluated pulmonary histological changes in some cases restricted their interest to the trachea or main bronchus [7,27,28]. Collins [12] provided results of histologic evaluations of 15 mice, with photographs that appear to show a complete response for acutely sensitized mice and an incomplete response for chronically sensitized mice. Because immunization is known to in and of itself be responsible in humans and cats for an anaphylaxis, [29,30] the findings suggest that any study comparing immunized and allergic mice carefully evaluate the histological findings in all animals to exclude those who experienced allergic pulmonary inflammation resulting from the immunization. Four sensitized animals in this study developed a complete histological response. The allergic response, if it occurred, occurred at an earlier stage is suggested by the absence of detectable IL-5 in three of the mice.

When histological findings are obliterated by therapy, the change of a histological response, and not the degree of the response, is documented. Soluble IL-15Rα [20] and rolipram [21] obliterate inflammation, extirpating, not simply diminishing, the response. Antigen pulsed dendritic cells alter the histological appearance by eliminating the eosinophils, which does not merely diminish the asthmatic attack, but actually reduces the airway responsiveness to methylcholine beneath that observed in control animals [22]; the latter paper is of note, as it identified as important interalveolar and subpleural inflammatory infiltrate. Desmet [23,24] showed diminution to an apparent incomplete response after treatment with nuclear factor- κB (NF-κB) and activator protein-1 decoy oligodeoxyribonucleotides. Photographs in this paper show peri-bronchial inflammation, the reason for the designation, in this paper, of "bronchoarterial space." Inflammation is not uniformly distributed about the airways, but shows a marked predilection for the side adjacent to arteries and arterioles. For this reason, statements such as "peri-bronchial and peri-vascular" [22,25] confuse unless the latter term references pulmonary veins. However, IL-4 and IL-13 were reported to induce airway responsiveness in the absence of inflammation [26].

de Siqueira [13] found wrinkling of the epithelium and dilatation of the alveolar spaces, which they demarcated as emphysematous to be of importance. Because their mice underwent BAL, it became of interest to see if the wrinkling and the alveolar dilatation might be unrelated to an asthmatic challenge. Some degree of bronchial epithelial wrinkling was seen in at least one airway in all mice; because quantifying the degree of wrinkling and the proportion of airways with wrinkling was difficult at best. This study concentrated on two relatively easily observed indications of lung injury not seen in every mouse--alveolar dilatation (denoted by these authors as emphysema) and hemorrhage. The results of the present study showed the latter two findings bore no relationship to stress, allergic challenge, IL-4, IL-5, the number of epithelial cells per 0.1 mm of basement membrane, or mitoses, meaning they likely reflected injury from BAL. Although such remodeling might be seen in mice that had not undergone BAL as a result of an allergic insult, the changes would be irrelevant to the determination of the presence or absence of histologic alterations resembling asthma after BAL; therefore we suggest studies of remodelling after BAL must take into account the potential for BAL-induced changes.

Brewer [8] included a grading system that evaluated the percentage of bronchioles involved. The promising results may have been in part due to its comparing mice that lacked an allergic/asthmatic response to mice that bore one; among all but the four mice strains who had both extremely low airway reactivity and inflammation, there appeared to be no relationship between the inflammatory scores and reactivity. The present study affirmed the diagnostic utility of the proportion of respiratory passages involved by inflammation. Pastva [17] devised a semi-quantitative inflammatory grading system that served as the basis of the one used in this study. We did not notice differences in cell height, which in the exercised/stressed group shown by their figure 1b[17] appeared similar to that seen in another published study of an unexercised mouse group [18]. Furthermore, it was unclear what the authors meant by degree of inflammation. Evaluation of the modified grading system showed no results that were inexplicable by chance, but did suggest the direction of the remaining grading studies. Because hematoxylin and eosin is not the best stain to evaluate mucin, any conclusion with respect to mucin is of limited value.

Different mouse strains and different allergic/asthma models yield different histological appearances [8,10-12]. For each mouse strain, a histological definition of the murine histologic alterations resembling asthma should be derived and verified. The current findings apply only to the BALB/c OVA mouse model. Strain of mouse and antigen/model selection will influence the inflammatory response and persistence of airway responsiveness [11].

Varying the time between last exposure and sacrifice decreases the acute response with time [6,31]. Molecular markers may also be of use; Duan [14,15] evaluated the effectiveness of p38α mitogen-activated protein kinase anti-sense oligonucleotide and U0126, a specific MAPK/ERK kinase inhibitor on inflammation as outcomes to evaluate a scoring system [16]. Concerning grading parameters, although the number of bronchial epithelial cells/mm^2 ^was slightly lower, consistent with the notion that larger cells were replacing smaller cells [32,33], the small size of the differences precludes their routine use. Image analysis is likewise time consuming, but does have the advantage of some objectivity. One study showed differences in the concentration of eosinophils [18] over time, as well as, with the use of immunohistochemistry, changes in smooth muscle thickness [33]. When time after stress was used as a means to provide a graded level of mediator, the proportion of respiratory passages showing inflammation and the presence of mitoses proved important, suggesting the possibility of their utility in a histological grading system. The histological score might be a better guide for the severity of allergic pulmonary inflammation than analysis of BAL fluid or chemical analyses of whole tissue sample. Eisenbarth [19] assessed the importance of differing concentrations of lipopolysacharide on the asthmatic response. Photographs of hematoxylin and eosin stained sections showed no difference with respect to the dosage of lipopolysaccharide, notwithstanding marked changes in the laboratory findings. In the grading criteria proposed here, the proportion of inflamed respiratory passages and mitoses, are of interest because they lie in opposition to one another. One fault of prior grading systems is the assumption that all findings are additive.

The two different stresses deserve further comment. Psychostimulants such as amphetamine and cocaine can induce a withdrawal syndrome minimally obfuscated by somatic signs, being mostly a psychological/neurochemical stressor [34]. Withdrawal from repeated amphetamine stimulation results in distress and depressive-like behaviors in rodent models [34,35]. In humans, the relatively transient withdrawal" or "distress" syndrome is characterized by depression, including psychomotor alterations, dysphoria, anxiety, anhedonia and anergia [34]. Post stimulants can also induce psychotic states in humans. In rats, repeated administration of psychomotor stimulants, such as amphetamine leads to behavioral sensitization and a progressive augmentation of behavioral responses to drug administrations, which persist even after long withdrawal periods [34,36]. Although the forced swim test would seem to simply be a acute physical stressor, it is also a model of the mental stress of depression [37-39] during acute stress, being a widely used pharmacologic model for assessing antidepressant activity in the rodent laboratory [35,39]. Researchers describe a "depressive" behavior as the floating response because the swimming behavior ceases and a state of despair or subjective helplessness is observed [36,38]. Neither form of stress proved important in any overall way that could not have been explained by chance, supporting the utility of the definition in animals that had undergone stress and animals that had not undergone stress. Because two amphetamine-stressed control animals developed a complete response and had detectable IL-5, relationship of stress to the induction of an acute allergic response must be further explored. Future analyses of allergic asthma might wish to quantify differences in BAL fluid with respect to inflammatory mediators and extensively analyze the effects of changes in immunization protocols to assess differences between mice that are physically stressed and mice that only experience psychological stress.

In another report by our laboratory, an analysis of mean levels of IL-4, but not IL-5, were elevated among mice exposed to the forced swim test compared to control mice[40] Thus, while control and non-allergic-challenged stressed mice did not differ in the proportion of mice with detectable IL-4 in BAL, the mean levels of IL-4 were elevated among stressed mice. Mice with histologic alterations resembling asthma responded to stress differently than did control mice, such that mean IL-4 levels did not differ between stressed and non-stressed allergic animals[40] By contrast, although mean IL-5 levels were elevated among allergic challenged animals, stress actually reduced mean IL-5 levels in BAL among allergic mice[40]

Limitations of this study include the absence of image analysis, special stains, gene expression analyses, and immunohistochemical studies, and lack of data on airway resistance. The definition and proposed grading system is limited to the BALB/c OVA mouse model. Similar studies should be undertaken for other mouse models before identifying a histologic alterations resembling asthma. There exists no reason a priori for levels of any specific inflammatory mediator in any specific mouse model to correlate with the degree of severity of an allergic challenge because inflammatory mediators may induce a response without a proportional histological response. The proposed grading system must be verified by a physiologic grading system that includes measures of air way resistance or pulmonary hyperresponsiveness. Despite these limitations, the value of the histological definition lies in the simplicity and speed with which the most commonly used murine model of allergic pulmonary inflammation can be confirmed. With practice, the identification of a response takes less than one minute and can be performed on animals that have little or no other measures of allergic pulmonary inflammation or asthma.

## Conclusion

A definition of murine histologic changes resembling asthma has been developed and verified. We describe and validate histological features previously designated as being specific to allergic pulmonary inflammation and to the injury induced by BAL. Because the histological constellation is unaffected by the presence or absence of stress and was validated in the presence of BAL cytokine changes, it should find utility in studies that use the BALB/c OVA mouse model. An allergic pulmonary inflammation grading system was also proposed.

## Methods

This work was approved by the Texas Tech University Animal Care and Use Committee prior to the start of work. For the initial study, 72 female BALB/c mice (6 to 8 weeks of age) were obtained from Charles Rivers Laboratories (Boston, MA); two mice were housed per bedded shoebox cage with a filter top. For the second study, 80 female BALB/c mice (8 weeks of age) were obtained from Jackson Laboratories (Bar Harbor, ME); two mice were housed per bedded shoebox cage with a filter top. Differences in the source of the animals related to purchasing arrangements of the animal care facility, but the animal strains were identical for all three study groups. For the third study, 25 female BALB/c mice (6 to 8 weeks of age) were obtained from Charles Rivers Laboratories (Boston, MA); five mice were housed per bedded shoebox cage with a filter top. Animals were maintained at 21-23°C with a 12:12 h light-dark cycle. Mice were provided an OVA free diet and water ad libitum. Animal protocols were approved by the Animal Care Committee of the Texas Tech University, which is accredited by AAALAC International. Animals were randomly assigned by cage to the different treatment and stress groups. The first study comprised six groups of twelve mice each, randomly assigned: 1) stressed, control; 2) stressed, sensitized; 3) stressed, challenged; 4) not stressed, control; 5) not stressed, sensitized; 6) not stressed, challenged. The second study comprised four groups of twenty mice each, randomly assigned: 1) stressed, control; 2) stressed, challenged (two of whom died); 3) not stressed, control; 4) not stressed, challenged. For the third study, challenged, not stressed mice were randomly assigned by cage to one of five time exposure treatments (0, 12, 24, 36, and 48 hours).

For the first study set, stress comprised intraperitoneal injections of amphetamine (d-amphetamine (AMP, Sigma-Aldrich, St. Louis, MO) 1 mg/kg, dissolved in saline less than one hour before injection)[34], on days 21, 22, and 23; mice who did not experience stress were provided intraperitoneal injections of sterile saline. For the second study set, stress comprised a 20 minute forced swim in a glass cylindrical jar, 27 cm tall, and 12 cm diameter, filled 15 cm with water maintained at 28-30°C [37]. Stress was induced on day 26 immediately prior to euthanization for the first two studies.

For the third study set, after the last exposure of aerosolized 1% OVA, mice were randomly assigned by cage to one of five time after exposure treatments (0, 12, 24, 36 and 48 h). For 0 and 12 h treatments, mice were exposed to aerosol OVA at 7:00 h and harvested at 7:20 h and 19:20 h respectively. For 24 and 36 h treatments, mice were exposed to aerosolized OVA at 7:30 h and harvested the next day at 7:50 h and 19:50 h respectively. For 48 h treatment mice were exposed to aerosol OVA at 8:00 h and harvested 48 h later at 8:20 h. The aerosol exposure and harvest times were allotted these times due to availability of equipment, although a stringent schedule was allotted to avoid variability from circadian effects

Immune activation was performed similarly to established allergy models [37,41]. On days 7, 14, and 21 sensitized and challenged mice received 10 μg of OVA (crude grade IV; Sigma, St. Louis, MO) i.p. emulsified in 100 μL of aluminum hydroxide gel (Sigma) and suspended in 100 μL of PBS [42]. Control mice received 200 μL of vehicle intra-peritoneally. Systemic levels of OVA-specific immunoglobulin (Ig)E were allowed to increase for 15 days after the fist sensitizing injection. On days 22, 23, 24, and 25, mice allocated to the challenged treatment group were exposed for 20 min to aerosolized OVA in PBS (1% w/v) generated with a nebulizer (Minimate Compressor PM, Precision Medical, Inc., North Hampton, PA) and mice in the control and sensitized treatment groups were exposed to aerosolized PBS for 20 min, a similar established method to previous studies [43], immediately before euthanization.

All mice chosen for the first two study sets were euthanized on day 26 using CO_2 _inhalation followed by thoracic decompression; mice chosen for the third study set were euthanized at time periods described above using using CO_2 _inhalation followed by thoracic compression. Immediately after euthanization, tracheas were exposed surgically and cannulated using an 18 g needle. Lungs were lavaged with 2 × 0.5 mL aliquots of ice cold PBS/2% fetal bovine serum (FBS). Supernatant was collected after centrifuging samples for 10 min at 1200 rpm and immediately frozen in a -70°C freezer. Multiple analyses were performed on the BAL fluid, blood, and serum, reported elsewhere (Sutherland, submitted); but the only measurements relevant to this effort were the IL-4 and IL-5 measurements performed BAL and IgE measurements performed on serum on the first study group when sufficient material was present. IL-4 and IL-5 levels were determined by by ELISA (Assay Designs, Ann Arbor MI), following manufacturer's guidelines, with sensitivity for IL-4 being 4.34 pg/ml and for IL-5 being 5 pg/ml. Plasma IgE concentrations were assayed at a 1:50 dilution by a commercially available ELISA kit (Bethyl Laboratories, Montgomery, TX) with a sensitivity of 3.9 ng/ml, according to the manufacturer's instructions.

Lungs were then surgically harvested and placed into 10% formalin for at least 5 days prior to paraffin embedding. Histologic analyses were performed on 4 μ thick parasagittal sections of whole lungs stained with hematoxylin and eosin. Totally embedded lungs of mice were evaluated for six changes deemed most important, as described in the results.. Second study set lungs were further examined for the presence or absence of alveolar dilatation and hemorrhage.

All mice from the first study with a complete response were selected for evaluation of a subjective inflammatory grading system. Pastva [17] had created a semi-quantitative system to evaluate the intensity of inflammation based on a series of subjective criteria, 1) bronchial inflammation; 2) vascular inflammation; 3) epithelial hyperplasia/hypertrophy; 4) goblet cells and mucin production; and 5) overall lung appearance. Initial attempts to distinguish overall bronchial from overall vascular inflammation were quite difficult, and Pastva [17] found neither a statistically significant criterion; the two criteria were fused into "bronchovascular inflammation." Although PAS staining was not performed, goblet cells were identified in all mice. Some form of hypertrophy/hyperplasia was also seen in virtually every mouse. For these reasons, each of the three categories, 1) bronchovascular inflammation, 2) epithelial hyperplasia/hypertrophy, and 3) goblet cell metaplasia/mucin production, was graded on a 1-4 scale, with 4 being the most severe and 1 being the least. Scores were multiplied by an overall severity score, graded from 1-4, and summed to yield an inflammation index. From the second study set were chosen 32 challenged mice with a complete histologic response and 32 control mice, none of whom had had a complete histologic response, for quantitative evaluation of two proliferation/hypertrophy indices: for each mouse the largest non-tracheal airway was evaluated at 200× for 1) the presence or absence of bronchial epithelial mitoses and 2) the number of epithelial cells counted over 0.1 mm of basement membrane in a flattened area. All mice in the third study set with a complete response were evaluated for two parameters: 1) the largest two non-tracheal airways were evaluated at 200× for the presence or absence of mitoses; 2) for all material on the slide, non-tracheal respiratory passages were evaluated at 100× for chronic inflammatory infiltrates and the number with and the number without inflammatory infiltrates were recorded.

Fisher's exact tests, two-tailed, evaluated contingency tables, with exact methods used to establish 95% confidence intervals (95% c.i.) for 2 × 2 tables. A Kruskal-Wallis tests, two-tailed, evaluated differences in medians. For univariate comparisons with a continuous outcome variable, simple linear regression analyses calculated point estimates and 95% c.i. of mean differences. For multivariate comparisons with a continuous outcome variable, log gamma regression calculated point estimates and 95% c.i. of mean ratios for continuous outcome variables. Binomial logistic regression analyses calculated adjusted odds ratios (OR) and 95% c.i. for multivariate analyses with dichotomous outcome variables and with analyses of dichotomous outcome variables with continuous predictor variables. Multinomial logit regression analyses calculated relative risks (RR) and 95% c.i. for categorical outcome variables other than dichotomous. Null hypotheses were rejected when *P *< 0.05.

## Authors' contributions

MSW evaluated the histologic specimens and performed the statistical analyses. GS participated in the design and coordination of the study and helped to draft the manuscript. MS performed the animal studies, participated in the design and coordination of the study and helped to draft the manuscript. JJM conceived of the studies, participated in its design and coordination, and helped to draft the manuscript. All authors read and approved the final manuscript.

## Supplementary Material

Additional file 1 **Characteristics of interest for the first study set**. The file contains frequency distritubions and univariate statistical analyses for the 72 mice in the first study set. Click here for file

Additional file 2 **Characteristics of interest for the second study set**. The file contains frequency distritubions and univariate statistical analyses, Fisher's exact test, two-tailed, for the 78 mice in the second study set that were used for analysis Click here for file

Additional file 3 **Proliferation parameter evaluation for 64 mice chosen from second set**. The file contains frequency distritubions and univariate statistical analyses, for the 64 mice from the second study set that were used to analyze epithelial cell concentration and mitotic activity. Click here for file
